# Prevalence and Characteristics of Neonatal Comfort Care Patients: A Single-Center, 5-Year, Retrospective, Observational Study

**DOI:** 10.3389/fped.2018.00221

**Published:** 2018-08-20

**Authors:** Lars Garten, Sjoukje Ohlig, Boris Metze, Christoph Bührer

**Affiliations:** Department of Neonatology, Charité – Universitätsmedizin Berlin, Berlin, Germany

**Keywords:** newborn, end-of-life care, palliative care, life-limiting, neonatal comfort care, circumstances of death, transition to home, NICU

## Abstract

**Objective:** To investigate the prevalence and characteristics of neonates with life-limiting or life-threatening conditions who receive care focused exclusively on comfort.

**Methods:**Retrospective chart review of all newborn infants admitted to a level III perinatal center within a 5 year period.

**Results:**1,777 of 9,878 infants (18.0%) had life-limiting or life-threatening conditions. 149 (1.5% of all neonates) were categorized as comfort care patients with death being anticipated within hours to weeks. 34.2% of comfort care patients suffered from conditions specific to the neonatal period, 28.9% were preterm infants at the limit of viability, and 22.8% were patients with congenital complex chronic conditions. In 80.5% of all comfort care patients treatment goals were re-directed toward a comfort-care-only regimen only once that life-prolonging therapies were demonstrated to be unhelpful. 136/149 comfort care patients (91.3%) died in hospital, while 13 (8.7%) were discharged home or into a hospice. Median age at death for comfort care patients was 3 days after birth (interquartile range 1–15.5 days), and delivery room death immediately after birth occurred in 37 patients (27.2%).

**Conclusions:** The vast majority of neonatal comfort care patients died in the hospital during the first week of life. However, almost one in 10 comfort care patients were discharged to home or hospice, suggesting that planning transition out of the NICU should be routinely discussed for all infants receiving comfort care.

## Introduction

Despite advances in both prenatal and neonatal care the largest subgroup of deaths in childhood are neonatal deaths (1, 2). Neonates die secondary to a wide variance in congenital complex conditions, acute conditions specific to the neonatal period, or complications of extreme prematurity in the face of rapidly increasing technology (3). For most newborn infants with life-limiting and life-threatening conditions comfort care measures are initialized in the neonatal intensive care unit (NICU) setting. In the developed world, if neonates die, in more than 90% of cases their terminal comfort care has been provided in a NICU (4). Surprisingly, there remains a paucity of reliable information regarding the prevalence and characteristics of NICU patients with life-limiting or life-threatening conditions who receive care focused exclusively on comfort.

Recent years have witnessed a significant increase in research focused upon children with life-limiting or life-threatening conditions, to describe the prevalence, diagnostic patterns and circumstances in which these children receive terminal comfort care (5–10). One way to calculate the prevalence of children who might require comfort care is to use a list of potentially life-limiting or life-threatening diagnoses. This approach is often used in palliative care studies, e.g., by Fraser et al. (8). Fraser et al. have used disease codes within a coding framework of the International Classification of Diseases, 10th Revision, to calculate the prevalence of children with life-limiting conditions in England by analyzing an Hospital Episode Statistics dataset (2000/2001–2009/2010) of 338.677 children (7, 8, 11). However, this approach turns a blind eye to the broad variety of disease courses that hide behind the same disease code in different patients. Furthermore, those lists are too broad to identify comfort care only patients because they include children who might live for years. This perspective may be particularly valid within the neonatal population. A newborn diagnosed genetically as having cystic fibrosis, for example, might live years without developing any challenging or complex symptoms. Such a child could initially be considered to have a life-limiting disease yet without receiving care focused exclusively on comfort for a long time. Accordingly, the definition of a comfort care patient has been called to be expanded to include elements of the patient's present health status.

The aim of this study was to describe neonates with potential life-limiting or life-threatening diagnoses who receive care focused on comfort in the NICU. We asked the following questions: (i) What is the prevalence of neonates with life-limiting or life-threatening conditions in a level III NICU? (ii) How many neonates with life-limiting or life-threatening conditions can be defined as “comfort care patients?” (iii) Is there a pattern of diagnoses among these infants? and (iv) Where and when are neonatal comfort care patients most likely to die?

## Methods

### Patients

The study population consisted of all neonates who were cared for in two level III neonatal intensive care units (NICU) of the perinatal center at the Charité University Medical Center (Berlin, Germany) between January 1, 2009 and December 31, 2013.

The two NICUs are located approximately 4 km apart from each other, but belong to a single Department of Neonatology, and use the same standard operating procedures. The same medical staff works in both units. Initial analysis of data from each unit indicates no significant differences in medical or sociological variables, staffing patterns, or patient characteristics. All eligible participants were identified by examining electronic hospital documentation.

For the purpose of this study we followed a two-step approach to identify comfort care patients. First, all patients with life-limiting or life-threatening conditions were identified by using a coding framework of ICD-10 disease codes (see Defining Life-Limiting and Life–Threatening Conditions). Second, we aimed to identify “comfort care patients.” We faced the dilemma that there is no uniformly accepted definition of a “comfort care patient,” but aimed for a definition that above all takes clinical relevant aspects into account (see Defining Comfort Care Patients). In addition, two separate classification systems were utilized to further analyse the comfort care patient population. We aimed for one classification system that is widely used in pediatric palliative care research and one system based on published modified classifications of pediatric/neonatal intensive care patients (see Defining Diagnostic Subgroups Among Comfort Care Patients).

### Defining life-limiting and life–threatening conditions

Diagnoses in the electronic file documentation system were coded according to the International Classification of Diseases, 10th Revision (ICD-10), disease classification (12). For the purpose of this study neonates with life-limiting or life-threatening conditions were identified using the coding framework of ICD-10 disease codes developed by Fraser et al. (8). The methodology developed by Fraser et al. calculated the prevalence of children with life-limiting conditions in the UK by applying a customized coding framework of the International Classification of Diseases, 10th Revision, disease codes to an English Hospital Episode Statistics dataset. The selection of ICD-10 codes to identify life-limiting conditions for this study was derived from two independent sources: (i) the Hain Dictionary version 1.0 of ICD-10 codes for children seen by palliative care providers and (ii) a listing of written diagnoses for children accepted for care at Martin House Children's Hospice between 1987 and 2010. The final ICD-10 coding framework consisted of 777 four digit ICD-10 codes. The diagnoses were categorized into 11 groups identical to the main ICD-10 chapters: neurology, hematology, oncology, metabolic, respiratory, circulatory, gastrointestinal, genitourinary, perinatal, congenital and other.

In order to more precisely characterize the neonatal cohort included in this study the framework of ICD-10 codes by Fraser et al. was modified by the addition of all infants with an extremely low birth weight below 500 g (ICD-10 code P07.00). Therefore, Fraser's category “perinatal,” was modified and further subdivided into two groups:

(i) Preterm infants at the limits of viability, with either a birthweight under 500 g or a gestational age between 22 0/7 and 23 6/7 weeks. These infants did not have another life-threatening illness.
(ii) Patients with diagnoses arising during, or specific to the neonatal period.

In addition, it was verified that all live-born patients with a gestational age of at least 22 0/7 weeks who died during the study period were included in this study group independent of their diagnosis. Thus, it was guaranteed that no patient who died in the delivery room (while receiving cardiopulmonary resuscitation or under primary comfort care), or in the neonatal intensive care unit (while receiving maximum treatment or after withholding or withdrawal of life support measures) was excluded.

### Defining comfort care patients

In order to define a patient as a “comfort care patient” all electronic files and medical reports of neonates identified with life-limiting or life-threatening conditions were screened for the existence of an advanced life threatening condition whose cure, stabilization or amelioration was no longer possible or desirable. Patients' medical charts were screened for written documentation of end-of-life decisions (e.g., “Do Not Attempt Resuscitation”- and/or “Allow Natural Death”-orders), or declared limitations (withholding or withdrawal) of at least one life-sustaining medical or surgical intervention indicated for immediate survival.

Both characteristics had to be present to define a patient as a “comfort care patient.”

### Defining diagnostic subgroups among comfort care patients

Two additional separate classification systems were utilized to further analyse the comfort care patient population in search of identifiable characteristics associated with neonatal comfort care patients. The first classification system used was based upon the four disease categories defined by the Association for Children's Palliative Care (the “Together for Short Lives (TfSL) classification,” formerly known as the “ACT classification”) (Table 1) (13). This classification proposes to identify pediatric patients for whom it would be beneficial to initiate specialized palliative care irrespective of disease stage and clinical complications.

**Table 1:** Together for Short Lives (TfSL) classification: disease categories of patients as defined by the Association for Children's Palliative Care.

**Category 1**	**Life-threatening conditions for which curative treatment may be feasible but can fail**. Access to palliative care services may be necessary when treatment fails or during an acute crisis, irrespective of the duration of threat to life. On reaching long-term remission or following successful curative treatment there is no longer a need for palliative care services. *Examples: extreme preterm birth, cancer, irreversible organ failure of heart, liver, kidney*.
**Category 2**	**Conditions where premature death is inevitable. Treatment may aim at prolonging life and allowing normal activities**. There may be long periods of intensive treatment aimed at prolonging life and allowing participation in normal activities. *Examples: cystic fibrosis, Duchenne muscular dystrophy*
**Category 3**	**Progressive conditions without curative treatment options**. Treatment exclusively palliative, may extend over years. *Examples: metabolic disorders, neuromuscular diseases*
**Category 4**	**Irreversible but non-progressive conditions causing severe disabilities leading to susceptibility to health complications and likelihood of premature death** *Examples: severe cerebral palsy, multiple disabilities such as following brain or spinal cord injury, complex health care needs, high risk of an unpredictable life-threatening event or episode*.

The second classification system used was based upon classifications of (neonatal) intensive care patients by Garten et al. (14), Stephens et al. (15), and Verhagen et al. (16):

(1) preterm infants at the limits of viability (gestational age between 22 0/7 and 23 6/7 weeks or birth weight below 500 g)
(2) patients with diagnoses with onset during or specific to the neonatal period (e.g., birth asphyxia, necrotizing enterocolitis/focal intestinal perforation/meconium ileus, hydrops fetalis, bronchopulmonary dysplasia, early/late onset sepsis, anhydramnion-associated lung hypoplasia, laparoschisis)
(3) patients with congenital complex chronic conditions
(4) others (further subdivided into the following subcategories: oncologic/hematologic, cardiovascular, neuromuscular, respiratory, renal, metabolic, gastrointestinal, and sudden infant death syndrome).

### Approval by ethics committee

The study was approved by the local institutional review board (Ethikkommission der Charité Universitätsmedizin Berlin, EA2/151/15) which waived the need to obtain informed parental consent.

### Statistical analysis

Patients' baseline characteristics are described as percentages (%) or median (interquartile range). Descriptive statistics consisted of frequency distributions of the variables. All statistical calculations employed SPSS 19.0 (SPSS Inc, Chicago, Ill).

## Results

Between January 2009 and December 2013, 9,878 neonates were cared for at one of the participating NICUs. We identified 1,777 neonates (18.0%) with a life-limiting or life-threatening condition as defined by the coding framework of ICD-10 disease codes used by Fraser et al. (8). This group of 1,777 patients included all live-born neonates with a gestational age of at least 22 0/7 weeks who died during the study period in our institution. Of all these patients cared for, 1.5% (149/9,878) were categorized as “comfort care patients.”

Differences regarding the distributions of diagnoses between neonates with life-limiting or life-threatening conditions and those neonates characterized as comfort care patients are summarized in Table 2.

**Table 2:** Differences between neonates with life-limiting or life-threatening conditions and neonatal comfort care patients.

	**Neonates with a life-limiting or life-threatening condition** ***N*** = **1,777**		**Neonatal comfort care patients** ***N*** = **149**	
Sex (female:male)	762:1,015		63:86	
Gestational age (weeks)*	37 0/7 (30 6/7–39 1/7)		30 4/7 (24 1/7–36 6/7)	
Birth weight (g)*	2,610 (1,430–3,320)		1,410 (585–2,500)	
Died before discharge from hospital (*n*)	213 (12.0%)		136 (91.3%)	
Age at death (days)*	3 (1–18)		3 (1–15.5)	
Discharged from hospital (*n*)	1,564 (88.0%)		13 (8.7%)	
- to hospice	2		2	
- to home, supported by a specialized pediatric palliative care team	5		5	
Age at discharge (days)*	21 (9–54)		21 (13–118)	
**Distribution on the main ICD10 chapters based on Fraser et al. (****8****)**	**All**, ***n*** **(%)**	**Died before discharge**, ***n***	**All**, ***n*** **(%)**	**Died before discharge**, ***n***
Neurology	12 (0.7%)	3	3 (2.0%)	3
Hematology	62 (3.5%)	3	1 (0.7%)	1
Oncology	19 (1.1%)	3	3 (2.0%)	3
Metabolic	64 (3.6%)	9	5 (3.6%)	5
Respiratory	80 (4.5%)	3	1 (0.7%)	1
Circulatory	16 (0.9%)	4	1 (0.7%)	1
Gastrointestinal	20 (1.1%)	3	2 (1.3%)	2
Genitourinary	18 (1.0%)	5	5 (3.4%)	5
SIDS	1 (0.1%)	1	–	–
Perinatal^#^	784 (44.1%)	149	94 (63.1%)	92
[preterm infants at the limit of viability (birthweight < 500 g or GA 22–24 weeks)]	[90 (5.1%)	57]	[43 (28.9%)	43]
[patients with diagnoses with onset in or specific for the neonatal period]	[694 (39.0%)	92]	[51 (34.2%)	49]
Congenital complex chronic conditions	701 (39.4%)	30	34 (22.8%)	23
All	1,777 (100.0%)	213	149 (100.0%)	136

*Data are presented as numbers (percentages) or median (IQR)*;

# *patients with diagnoses with onset during or specific to the neonatal period (e.g., birth asphyxia, necrotizing enterocolitis/focal intestinal perforation/meconium ileus, hydrops fetalis, bronchopulmonary dysplasia, early/late onset sepsis, anhydramnion-associated lung hypoplasia, laparoschisis)*.

213 of 9,878 neonates (2.2%) admitted to the NICUs died before discharge. Within the subgroup of neonates with life-limiting or life-threatening diseases the mortality rate was 12.0% (213/1,777). Of the 213 patients who died, 77 (36.2%) died while receiving support measures (including cardiopulmonary resuscitation), and 136 (63.8%) died under primary comfort care or after withholding or withdrawal of life support measures. Deaths in the delivery room shortly after birth accounted for 2.4% (43/1,777) of the total mortality in the subgroup of neonates with life-limiting diseases.

Within the neonatal comfort care patient group 136/149 patients (91.3%) died in hospital. 13/149 comfort care patients (8.7%) were discharged home or to a hospice. Median age at death in this group was 3 days (IQR 1–15.5 days).

Deaths in the delivery room shortly after birth accounted for 27.2% (37/136) of the total mortality in this group, all 37 neonates receiving immediate primary comfort care after birth.

**Figure 1:**
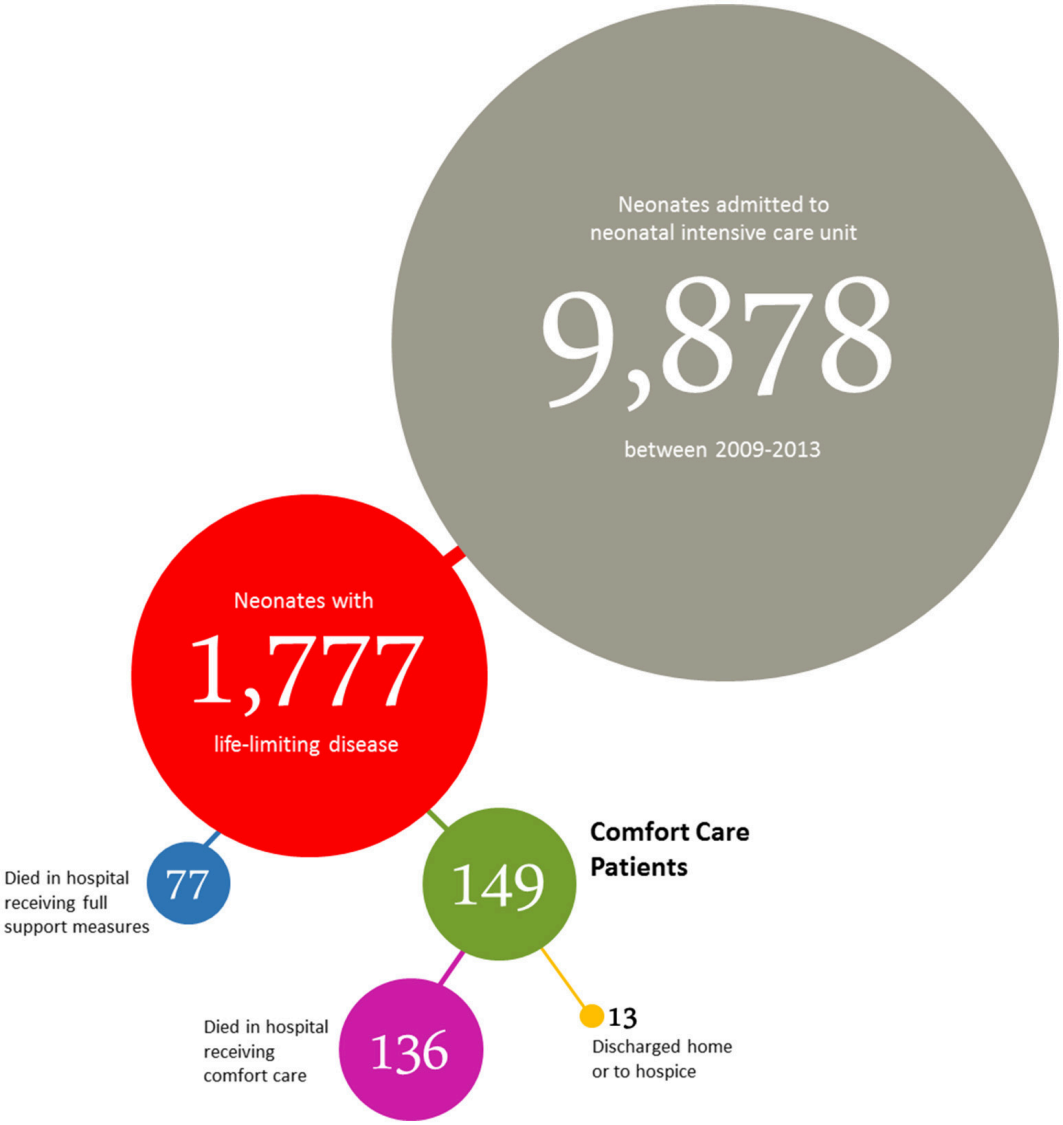
Distribution of neonates admitted to the NICUs of the perinatal center at the Charité University Medical Center (Berlin, Germany) between January 2009 and December 2013.

The distribution of study population to all subgroups is summarized in Figure 1.

The distribution of neonates with life-limiting or life-threatening conditions is detailed in Table 3A. The distribution of comfort care patients according to the modified classification of (neonatal) intensive care patients is shown in Table 3B.

**Table 3A:** Distribution of all neonates with life-limiting or life-threatening conditions [as defined by Fraser et al. (8)] cared for between 2009 and 2013.

**Neonates with life-limiting or life–threatening conditions** ***n*** = 1,777			
**Group 1:** Preterm infants at the limit of viability (birthweight < 500 g or GA 22–24 weeks)	**Group 2:** patients with diagnoses with onset in or specific for the neonatal period	**Group 3:** patients with congenital complex chronic conditions	**Group 4:** others
5.1% (*n* = 90)	39.1% (*n* = 694)	39.4% (*n* = 701)	16.4% (*n* = 292)
Died before discharge (*n* = 57) Age at death: 1 day (1–6 days)^*^	Died before discharge (*n* = 92) Age at death: 3 days (1.5–9.5 days)^*^	Died before dsischarge (*n* = 30) Age at death: 4 days (1–26 days)^*^	Died before discharge (*n* = 34) Age at death: 31 days (5–50 days)^*^
Discharged (*n* = 33) Age at discharge: 108 day (102–118 days)^*^	Discharged (*n* = 602) Age at discharge: 24 days (11–55 days)^*^	Discharged (*n* = 671) Age at discharge: 12 days (6–24 days)^*^	Discharged (*n* = 258) Age at discharge: 58.5 days (30–90 days)^*^

* *Data are presented as median and IQR*.

**Table 3B:** Distribution of all neonatal comfort care patients cared for between 2009 and 2013.

**Neonatal comfort care patients** ***n*** = 149			
**Group 1:** Preterm infants at the limit of viability (birthweight < 500 g or GA 22–24 weeks)	**Group 2:** patients with diagnoses with onset in or specific for the neonatal period	**Group 3:** patients with congenital complex chronic conditions	**Group 4:** others
28.9% (*n* = 43)	34.2% (*n* = 51)	22.8% (*n* = 34)	14.1% (*n* = 21)
Died before discharge (*n* = 43) Age at death: 1 day (1–3 days)^*^	Died before discharge (*n* = 49) Age at death: 3 day (2–9 days)^*^	Died before discharge (*n* = 23) Age at death: 4 days (1–25 days)^*^	Died before discharge (*n* = 21) Age at death: 33 days (10–52 days)^*^
Discharged (*n* = 0)	Discharged (*n* = 2) Age at discharge: 3 days; 19 days	Discharged (*n* = 11) Age at discharge: 44 days (13–155 days)^*^	

* *Data are presented as median and IQR*.

Among all neonatal comfort care patients 80.5% suffered from “life-threatening conditions for which curative treatment may be feasible but can fail” (TfSL category group 1). The detailed distribution of all comfort care patients according to the TfSL categorization scheme is shown in Table 4.

**Table 4:** Distribution of all analyzed neonatal comfort care patients according to the “Together for Short Lives (TfSL) classification.”

**Disease category according to the “Together for Short Lives (TfSL) classification”**	**Category 1** Life-threatening conditions for which curative treatment may be feasible but can fail.	**Category 2** Conditions where premature death is inevitable. Treatment may aim at prolonging life and allowing normal activities.	**Category 3** Progressive conditions without curative treatment options. Treatment exclusively palliative, may extend over years.	**Category 4** Irreversible but non-progressive conditions causing severe disabilities leading to susceptibility to health complications and likelihood of premature death
*N* (%)	120 (80.5%)	9 (6.0%)	18 (12.1%)	2 (1.3%)
Died before discharge (*n*)	116	7	12	1
Age at death (days)^*^	2 (1–10.5)	25 (7–62)	18 (4–39.5)	30

* *Median und IQR*.

## Discussion

This study retrospectively analyzes data of all neonates cared for in the level III Perinatal Center of the Charité Medical Center in Berlin during the 5 year period from 2009 to 2013. Approximately every fifth neonate cared for in the NICUs suffered from a “life-limiting or life-threatening condition” as defined by a modified coding framework of the International Classification of Diseases, 10th Revision, disease codes (8).

Within the study population 1.5% fulfilled the criteria of a “comfort care only” patient. One third of comfort care patients suffered from conditions whose onset developed within the neonatal period or were conditions specific to the neonatal period. About one quarter were preterm infants at the limit of viability, and one fifth were patients with congenital complex chronic conditions. Median age at death was 3 days, and one third of all comfort care patients died soon after birth while still in the delivery room. In more than 90% comfort care patients died in the hospital setting, therefore, the provision of comfort care to neonates was commensurate to providing “end-of life care” in the hospital setting.

Many physicians working in the field of pediatric palliative care have called for more frequent utilization of home or hospice care services by hospital physicians caring for newborn patients under comfort care only (17–20). Lower referral rates for neonatal comfort care patients to out-of-hospital palliative care services compared to referral rates for pediatric patients in other specialties—for example, those suffering from hemato-oncologic diseases (21, 22)—have been interpreted to suggest a negative attitude by neonatologists toward palliative and comfort care at home, respectively (23). In one study including both numerical and descriptive analysis of newborn children experiencing lengthy (>6 months) hospitalization, Catlin (24) reported a perception by NICU staff that the children's length of stay was being influenced by physicians' insistence on continued medical treatment despite evidence to suggest its futility. The author suggested that the medical orientation of NICU care providers may be a common barrier to the optimal use of family-centered palliative care (24).

Indeed, most discussions in the literature expressing dissatisfaction with the discharge rates of neonates being cared for under comfort care only to palliative and comfort care at home, respectively, have focused upon impediments posed by health care providers, administrative issues or environmental conditions (25–28).

In contrast, the data presented here suggest that the limited options for the subgroup of neonatal patients cared for under comfort care only could also reflect the special characteristics of this group of patients. The degree of prognostic uncertainty that accompanies the diagnosis of a life-limiting or life-threatening disease in neonatal patients is disproportionately far greater than that encountered in other palliative care populations. In our study, for example, 80.5% of all neonatal comfort care patients suffered from life-threatening conditions for which curative treatment might be feasible but could fail (TfSL category 1). Accordingly, most neonates finally designated as “comfort care patients” were supported initially by medical treatment with a curative goal. Treatment goals were re-directed toward a comfort-care-only regimen only once that treatment had failed. This time span was rather short, and several studies have shown that most neonatal deaths follow closely upon the withdrawal of life-sustaining treatment (29–31). A prospective observational study by Hellmann et al. (32) showed that after withdrawal of life-sustaining treatment in neonates, the median time to death was 1 h and the median age at death was 5 days (32). This report is consistent with our own work reported here, with a median time to death of 3 days after birth for a neonate defined as a comfort care patient.

We therefore propose that the high rate of neonatal comfort care patients dying in the hospital setting does not reflect the unwillingness of neonatologists to utilize hospice services *per se*, but rather reflects the specific circumstances of living and dying of infants admitted to neonatal care. Furthermore, caution is warranted also not to uncritically equate “home care” with “good care” or even “good death.” Despite its technological backdrop, the NICU setting may nevertheless be an environment that assures comfort and safety, a place with a familiar structure and staff providing emotional support, a place where quality-of-life considerations are an important part of decision making (33, 34). The NICU may be all of these, predicated on the assumption that the provision of high-quality comfort and palliative care is a fundamental goal embraced by all members of the NICU team (35). Indeed, the provision of cure-oriented, disease modifying medical treatment does not preclude the simultaneous co-administration of palliative care services, with the balance between the two being re-adjusted as the circumstances dictate (36).

However, there was a notable proportion of almost one in ten comfort care patients who was discharged to home or hospice. Based on this finding we suggest that planning transition out of the NICU should be routinely discussed for all children receiving comfort care. It should be the role of health professionals to discuss with families all options that are available, for families to determine what they would like and can manage, and for professionals to work with them to achieve their preference. Families should be allowed to make choices for their children and themselves. Some will choose to leave the neonatal unit, but others will stay, with familiar staff in an environment in which they feel safe and supported. Taking this striking aspect of the study into account the data presented here could redound to the benefit of parents wishing for home care for their child by motivating NICU staff to consider out of hospital care routinely. Even in challenging “comfort care only”- and “end-of life-care”-situations.

There are two main limitations to this study. First, we present data from a single center and cannot be sure that our findings are applicable to the entire population outside of our institution. Second, the data analysis of the deceased patients was retrospective, and documentation bias is difficult to control for in such a setting. All presented data depend on moderate quality of documentation. In some cases, entries were incomplete or illegible, or the desired information was not documented explicitly.

## Conclusions

In this study, one of every five neonates cared for at a level III perinatal center suffered from a life-limiting or life-threatening condition, yet only 1.5% fulfilled the criteria of a “comfort care only” patient. In most cases care for neonatal comfort care patients was essentially “end-of life-/terminal comfort care” in the hospital setting. Our work indicates that the high proportion of neonatal deaths occurring within a hospital setting presumably reflect patient- and disease-related issues more than a general unwillingness of NICU staff to offer choice of non-hospital care. However, almost one in 10 comfort care patients was discharged to home or hospice, justifying that planning transition out of the NICU should be routinely discussed for all infants receiving comfort care.

## Author contributions

LG, SO, and CB conceptualized the study. LG and SO collected the data. LG, SO, BM, and CB analyzed the data. LG wrote the first draft of the manuscript. All authors contributed to the interpretation of the data and critically reviewed and contributed to the final draft of the manuscript.

### Conflict of interest statement

The authors declare that the research was conducted in the absence of any commercial or financial relationships that could be construed as a potential conflict of interest.

## Abbreviations

ACT: Association for Children's Palliative Care
ICD-10, International Classification of Diseases: 10th Revision
NICU: neonatal intensive care unit.
